# Identification and treatment of viral hepatitis C in persons who use drugs: a prospective, multicenter outreach study in Flanders, Belgium

**DOI:** 10.1186/s12954-021-00502-7

**Published:** 2021-05-17

**Authors:** Dana Busschots, Cécile Kremer, Rob Bielen, Özgür Muhammet Koc, Leen Heyens, Eefje Dercon, Rita Verrando, Tessa Windelinckx, Griet Maertens, Stefan Bourgeois, Niel Hens, Catharina Matheï, Geert Robaeys

**Affiliations:** 1 Faculty of Medicine and Life Sciences, Hasselt University, Martelarenlaan 42, 3500 Hasselt, Diepenbeek, Belgium; 2 Department of Gastroenterology and Hepatology, Ziekenhuis Oost-Limburg, Genk, Belgium; 3 Interuniversity Institute for Biostatistics and Statistical Bioinformatics (I-Biostat), Data Science Institute, Hasselt University, Diepenbeek, Belgium; 4 School of NUTRIM, Maastricht University Medical Centre, Maastricht, The Netherlands; 5 zorGGroep Zin Limburg, Hasselt, Belgium; 6 Free Clinic Ngo, Antwerp, Belgium; 7 Harm Reduction, Coordinator GIG – Health promotion in Injecting Drug use Flanders, Flanders, Belgium; 8 Department of Gastroenterology, ZNA Antwerp, Antwerp, Belgium; 9 Centre for Health Economic Research and Modelling Infectious Diseases, Vaccine and Infectious Disease Institute, University of Antwerp, Antwerp, Belgium; 10 Department of Public Health and Primary Care, KU Leuven, Leuven, Belgium; 11 Department of Gastroenterology and Hepatology, University Hospitals KU Leuven, Leuven, Belgium

**Keywords:** Hepatitis C virus, Outreach, People who use drugs, Linkage to care, High-income country

## Abstract

**Background:**

Targeted screening for hepatitis C viral (HCV) infection is not yet widely executed in Belgium. When performed in people who use drugs (PWUD), it is mainly focused on those receiving opiate agonist therapy (OAT). We wanted to reach out to a population of difficult to reach PWUD not on centralized OAT, using non-invasive screening as a bridge to re-integration in medical care supported by facilitated referral to a specialist.

**Methods:**

This was a prospective, multicenter cohort study in PWUD not enrolled in a centralized OAT program in a community-based facility in Limburg or OAT program in a community-based facility in Antwerp, Belgium, from October 2018 until October 2019. Two study teams recruited participants using an outreach method at 18 different locations. Participants were tested for HCV antibodies (Ab) by finger prick, and risk factors were assessed through a face-to-face questionnaire. Univariate analyses were used to assess the association between HCV Ab and each risk factor separately. A generalized linear mixed model was used to investigate the association between the different risk factors and HCV.

**Results:**

In total, 425 PWUD were reached with a mean age of 41.6 ± 10.8, and 78.8% (335/425) were men. HCV Ab prevalence was 14.8% (63/425). Fifty-six (88.9%) PWUD were referred, of whom 37 (66.1%) were linked to care and tested for HCV RNA. Twenty-nine (78.4%) had a chronic HCV infection. Treatment was initiated in 17 (58.6%) patients. The adjusted odds for HCV Ab were highest in those with unstable housing 6 months before inclusion (*p* < .001, AOR 8.2 CI 95% 3.2–23.3) and in those who had ever shared paraphernalia for intravenous drug use (*p* < .001, AOR 6.2 CI 95% 2.5–16.0).

**Conclusions:**

An important part tested positive for HCV. Treatment could be started in more than half of the chronically infected referred and tested positive for HCV-RNA. Micro-elimination is necessary to achieve the World Health Organization goals by 2030. However, it remains crucial to screen and link a broader group of PWUD to care than to focus solely on those who inject drugs.

*Trial registration*: clinicaltrials.gov NCT04363411, Registered 27 April 2020—Retrospectively registered. https://clinicaltrials.gov/ct2/show/NCT04363411?term=NCT04363411&draw=2&rank=1

**Supplementary Information:**

The online version contains supplementary material available at 10.1186/s12954-021-00502-7.

## Background

The majority of chronic liver disease and liver-related deaths worldwide are caused by viral hepatitis infections [1, 2]. Concerning hepatitis C viral infections (HCV), the World Health Organization (WHO) has set targets to eliminate HCV by reducing new infections by 90% and mortality by 65% in 2030 [1, 3]. The prevalence of chronic HCV infection was estimated to be 0.12% among the general Belgian population in 2019 and is generally low (< 0.6%) in high-income countries [4–6]. Nonetheless, in high-risk populations such as people who use drugs (PWUD), HCV prevalence is increased [7]. In Ireland and Madrid (Spain), 50% and 33% of the PWUD were exposed to an HCV infection [8, 9]. A recent study estimated the HCV antibody (Ab) prevalence in Belgian people injecting drugs (PWID), a subpopulation of PWUD, at 41.1% [10]. However, no data on HCV Ab prevalence in the more general PWUD population are available for Belgium [11].

With direct-acting antiviral therapy and its ≥ 95% cure rate, HCV elimination is achievable [12, 13]. Between 2015 and 2017, direct-acting antiviral (DAA) treatment was only reimbursed if fibrosis was staged ≥ F3 in Belgium [14]. As of January 2017, the reimbursement criteria have been adjusted to ≥ F2. Unlimited access has only been possible since 2019 [15, 16]. However, DAA treatment can only be prescribed and initiated by a hepatologist and is available only in hospital pharmacies.

A Belgian ‘Hepatitis C Plan’ was developed in 2014 with the purpose to (1) reduce transmission, (2) increase the number of HCV-positive patients aware of their diagnosis, and (3) enhance the patient care pathway and quality of life [17]. However, to date, all efforts remain dependent on local initiatives and no strategy is implemented at a national level.

In Belgium, both NGO Free Clinic Antwerp and the zorGGroep Zin Limburg are community-based facilities specialized in addiction care and closely involved in HCV care for PWUD [11, 18]. They provide HCV care to PWUD enrolled in their drug services, opiate agonist treatment (OAT) program and/or needle syringe programs (NSP). However, young injectors who have not yet contacted these centers, former PWUD, stimulants injectors, and opioid users receiving OAT at their local pharmacy are often not reached [18]. Providing HCV care to these specific high-risk subgroups, who are at the heart of the epidemic, is challenging [3]. There are no good estimates of the size of these subgroups in Europe, which are often completely isolated from care. Nevertheless, outreach methods to contact vulnerable populations have been proven successful in different European countries and Australia [19–23]. We aimed to reach out to this specific and vulnerable PWUD community not reached within the OAT program of a community-based facility using a non-invasive screening method. Determining the prevalence of HCV Ab could give us a better idea of the current challenges concerning HCV among this difficult-to-reach high-risk group in a high-income country.

## Methods

### Study setting

The NGO Free Clinic in Antwerp and the zorGGroep Zin Limburg are community-based facilities for addiction care. Free Clinic is located in the city of Antwerp and zorGGroep Zin has several locations in the province of Limburg. Harm reduction such as OAT and NSP is offered in these centers in a very low-threshold manner. Clients can obtain OAT in two different ways at these centers. On the one hand, there is the on-site provision (centralized OAT) where the medication is taken at the center under supervision. On the other hand, a client may obtain a prescription from the attending physician to receive the medication from a local pharmacist.

Both centers offer HCV care (e.g., education and screening, extra support) to their clients.

All clients of the Free Clinic are offered and reached for annual HCV screening. Therefore, only individuals who were not registered at the MSOC (medical social center) Free Clinic were included in this study.

The zorGGroep Zin also offers a yearly HCV screening to all its clients. Clients on central provision are easily reached. However, previous research at zorGGroep Zin showed that clients who only receive an OAT prescription and receive their medication at the pharmacy are difficult to reach and are often not tested (data not shown). Therefore, this study only included individuals who were not registered at zorGGroep Zin or clients of zorGGroep Zin who only received their prescription at the center resulting in them not being reached for HCV care.

### Study design

#### Sample

This is a prospective, multicenter cohort study in PWUD. Participants were eligible for inclusion if they were aged 18 years or older, not enrolled in a centralized OAT program in Limburg or any OAT program in Antwerp from October 2018 until October 2019. PWUD were defined as people who have a history of drug use or who actively use drugs (excluding alcohol).

#### Intervention

The team in Antwerp consisted of a HCV reference nurse accompanied by a social worker, an addiction care physician, and peer workers. In Limburg, the team consisted of an HCV case manager nurse accompanied by a Ph.D. student and a medical Ph.D. student. The two teams recruited participants using the same outreach method by organizing screening events in 18 different locations across the city of Antwerp, the Kempen region, and the province of Limburg. The events’ locations were always communicated in advance, using posters and flyers to inform possible candidates. Locations existed of homeless shelters, local pharmacies involved in OAT care and NSP, addiction care centers not providing (centralized) OAT, and public locations (e.g., public squares, night shelters, low-threshold facilities of civil society organizations). The screening events were always organized in cooperation with peers, general physicians, the addiction care centers, and the Public Centre for Social Welfare. In the province of Limburg, the study team used a mobile home in several locations.

Participants were tested for HCV Ab by finger prick using OraQuick®. While waiting for the rapid test results (15 min), an encoded questionnaire on paper was filled out face-to-face in a private and secure setting. After completing the questionnaire and finalizing the tests, the participant was provided with a ten euro remuneration for participating in the study. Every participant was informed about all aspects of the disease, from the transmission to treatment. Additionally, if the test was positive, the study team took ample time to report the diagnosis and discuss it in detail and an appointment at the hepatology department was planned immediately. If the PWUD claimed to have been treated in the past, the research team contacted the specialist. If the specialist confirmed a successful treatment and a permanent HCV sustained virologic response without ongoing risk behavior, the PWUD was not referred further. In case of referral and when requested, the nurse and/or a peer accompanied the PWUD to the specialist’s appointment. During the screening, a telephone number or email address was requested from each PWUD who tested positive for a finger prick test. This telephone number (phone call/text) or email address was used to remind the PWUD at least three times of the appointment with the specialist in the hospital. Loss to follow-up (LTFU) was defined as loss of contact with the PWUD despite at least three contact attempts. Follow-up data after a positive finger prick test were retrospectively collected after 6 months. The data were collected by the nurses of both research teams based on pre-defined questionnaires.

The study was approved by the Ethical Committee of Ziekenhuis Oost-Limburg and Hasselt University (18/0052U). The study protocol is registered at clinicaltrials.gov (NCT04363411). The study was conducted in accordance with the provisions of the Declaration of Helsinki and its amendments. Good clinical practice guidelines were followed throughout the study, and all participants provided written informed consent [24].

#### Questionnaire

The questionnaire was available in Dutch, French, and English and covered a total of 22 questions. Data from the questionnaire included birth gender, year of birth that was categorized into: < 1955, 1955–1974, and > 1974 based on the European baby-boom cohort [25], source of income, level of education, housing past 6 months, ever have been incarcerated, alcohol abuse (> 14 units women or > 21 units men per week), age of first drug use, kind of drugs (ever, past 6 months), manner of drug use, frequency of drug use (past 6 months), kind of intravenous (IV) drugs (ever, past 6 months), frequency of IV drug use (past 6 months), sharing IV-related paraphernalia (ever, past 6 months) receiving OAT, connected to an NSP.

Follow-up data included: METAVIR score, the result of HCV RNA viral load, the HCV genotype, initiation of treatment, and the reason for not initiating treatment. METAVIR score is used to grade fibrosis in patients with HCV ranging from no fibrosis (*F*0) to cirrhosis (*F*4) [26]. Cut-off values for HCV as measured by FibroScan® are *F*0–*F*1 =  < 7.2 kPa, *F*2 = 7.2–9.5 kPa, *F*3 = 9.5–12.5 kPa, *F*4 =  > 12.5 kPa [27].

### Endpoints of the study

This study’s primary objective was to assess the HCV Ab prevalence using a rapid test in a high risk, difficult to reach subgroup of PWUD in Flanders, Belgium. Measuring the prevalence could give us a better impression of the current challenges concerning HCV in these PWUD. The secondary objective was to evaluate the linkage to care of PWUD who tested positive for HCV Ab.

### Statistical analyses

Patient demographics were summarized using mean ± standard deviation for continuous characteristics and by proportions for categorical characteristics. To assess differences in participant characteristics between the trial sites, the Chi-squared test or Fisher’s exact test was used for categorical variables and independent-samples *t* test for continuous variables.

Univariate models were used to assess the association for each risk factor separately. Risk factors associated (*p* < 0.150) with HCV Ab in these univariate analyses were included as fixed effects in a multiple GLMM. To account for heterogeneity between individuals from the different trial sites (Antwerp as urban and Limburg as mixed urban–rural), a generalized linear mixed model (GLMM) was used to investigate the association between the different risk factors and HCV Ab. In these models, the trial site was then included as a random intercept.

#### Sample size

The ideal sample size for a prevalence study is a function of the expected prevalence and precision for a given confidence level [28]. For a small prevalence, as is the case for HCV Ab, a conservative choice for the amount of precision has to be made using one-fifth of the estimated prevalence (for the effect size) [29].

In this study, an estimated HCV Ab prevalence of 30% was used. This is less than the estimated prevalence of HCV Ab in PWID [10, 11], but we chose this conservative estimate, as we also included non-PWID. With a confidence interval (CI) of 95%, *z* is 1.96. *P* is 0.30 and *d* = 0.30/5 = 0.06 (the formula is provided in [see Additional file 1]), where *z* is the quantile of the normal distribution corresponding to the level of confidence, *P* is the expected prevalence, and *d* is the effect size (i.e., the maximum difference between estimated and true prevalence). Therefore, an inclusion of 224 PWUD was necessary. However, since the prevalence will be estimated in a study using data from two sites (cluster design), the design factor was taken into account [30]. Therefore, the sample size was multiplied by a factor of 1.5 [30]. A total of at least 336 PWUD had to be included.

## Results

Between October 2018 and October 2019, 36 screening days at 18 different locations were organized. In total, 425 PWUD not connected to any OAT in Antwerp or the centralized OAT program in Limburg were reached. The socio-demographic characteristics of the study population are shown in Table 1. The population of Antwerp and Limburg did not differ in terms of age at inclusion (42.1 ± 10.7 vs. 41.0 ± 11.0, *p* = 0.280), gender (male: 81.2% vs. 76.6%, *p* = 0.270), age of first drug use (18.4 ± 8.0 vs. 18.3 ± 8.3, *p* = 0.906), ever injected drugs (yes: 32.9% vs. 36.7%, *p* = 0.394), or enrolment in an OAT program not linked to the center in Antwerp or the centralized program in Limburg (100% vs. 96.3%, *p* = 0.137). Therefore, the results of both locations are analyzed together.

**Table 1:** Baseline characteristics of the study population

Characteristics (*n* = 425)	*N* (%)
Age (years) mean ± SD (range)	41.6 ± 10.8
Gender (male)	335 (78.8)
Country of birth	
Belgium	333 (78.4)
Other	91 (21.4)
Missing	1 (0.2)
Source of income last 6 months	
Employment	87 (20.5)
Welfare check	254 (59.8)
Pension	13 (3.1)
None	68 (16.0)
Missing	3 (0.7)
Housing last 6 months	
At home (owned/rented)	225 (52.9)
Residential/family/friends	122 (28.7)
Prison/homeless	78 (18.4)
Missing	2 (0.5)
Level of education	
Primary school (7–12 years)	22 (5.2)
Partly completed high school (< 16 years)	130 (30.6)
Completed high school (18 years)	206 (48.4)
Higher education (> 18 years)	66 (15.5)
Missing	1 (0.2)
Ever have been incarcerated (yes)	227 (53.4)
Alcohol abuse	
Active (< 6 m)	122 (28.7)
Former	126 (29.6)
Never	175 (41.2)
Missing	2 (0.5)
Age of first drug use (years) mean ± SD (range)	18 ± 8.1
Ever IV drug use (yes)	148 (34.8)
Recent (< 6 m) IDU (*n* = 148)	73 (49.3)
Recently (< 6 m) injected not connected to NSP (*n* = 73)	13 (17.8)
Recently (< 6 m) used opioid not connected to OAT (*n* = 97)	47 (48.5)

*IV* intravenous, *OAT* opiate agonist therapy, *NSP* needle syringe program

### Prevalence HCV Ab

Sixty-three (14.8%) PWUD tested positive for HCV Ab using a finger prick test. Of them, 26 (41.2%) were unaware that they had ever been in contact with HCV and might have been infected. Looking only at the PWID in this population, 58/148 (39.2%) was HCV Ab positive.

### Risk factors associated with HCV Ab positivity

The unadjusted odds for HCV Ab were highest in those who had injected in the past 6 months before inclusion (*p* < 0.001, OR 31.3 CI 95% 14.1–77.5). Persons who were part- or full-time employed had a significantly lower odds of HCV Ab positivity compared to those that were unemployed (*p* = 0.014, OR 4.8 CI 95% 1.4–18.6) or received an allowance (*p* = 0.006, OR 4.5 CI 95% 1.7–15.3). The results of the univariate GLMM are provided in an additional file [see Additional file 2].

The random intercept variance for the trial site was estimated to be 0, indicating no difference between the trial sites in HCV Ab positivity. This was also shown in univariate analyses using a Chi-squared test (*p* = 0.063).

The adjusted odds ratio (AOR) for HCV increased significantly in PWUD (Table 2), who spent the last 6 months before inclusion in prison or were homeless (*p* < 0.001, AOR 8.2 CI 95% 3.2–23.3), who had ever shared paraphernalia for IV drug use (*p* < 0.001, AOR 6.2 CI 95% 2.5–16.0), who had used heroin in the last 6 months (*p* = 0.010, AOR 3.1 CI 95% 1.3–7.5), who had ever injected heroin (*p* = 0.001, AOR 5.1 CI 95% 2.0–13.6), who had injected amphetamines in the last 6 months (*p* = 0.002, AOR 4.6 CI 95% 1.8–12.2), or injected cocaine in the last 6 months (*p* = 0.010, AOR 4.9 CI 95% 1.5–17.4). On the other hand, having used cocaine in the last 6 months in general significantly decreased the odds of HCV (*p* = 0.005, AOR 0.2 CI 95% 0.1–0.6).

**Table 2:** Results of the multiple generalized linear mixed models

Risk factor	Estimate (SE)	*p* value	AOR (95% CI)
(Intercept)	− 4.122 (0.450)		
Housing last 6 m			
Owned/rented	(Ref)	(Ref)	
Residential/family/friends	0.686 (0.486)	.159	1.985 (0.759–5.193
Prison/homeless	2.115 (0.506)	**< .001**	8.201 (3.164–23.306)
Sharing paraphernalia			
Never	(Ref)	(Ref)	
Ever	1.830 (0.470)	**< .001**	6.231 (2.514–16.042)
Last 6 months	− 0.948 (0.700)	.175	0.388 (0.094–1.477)
Drug use last 6 m—cocaine	− 1.406 (0.501)	**.005**	0.245 (0.088–0.626)
Drug use last 6 m—heroin	1.333 (0.441)	**.010**	3.105 (1.317–7.464)
IV drug use ever—heroin	1.635 (0.486)	**.001**	5.127 (2.004–13.572)
IV drug use last 6 m—amphetamines	1.524 (0.492)	**.002**	4.593 (1.753–12.219)
IV drug use last 6 m—cocaine	1.597 (0.619)	**.010**	4.939 (1.515–17.370)

The **p**-values indicated in bold are significant values

*AOR* adjusted odds ratio, *CI* confidence interval, *IV* intravenous, *ref* reference

### Linkage to care and treatment for HCV

**Fig. 1:**
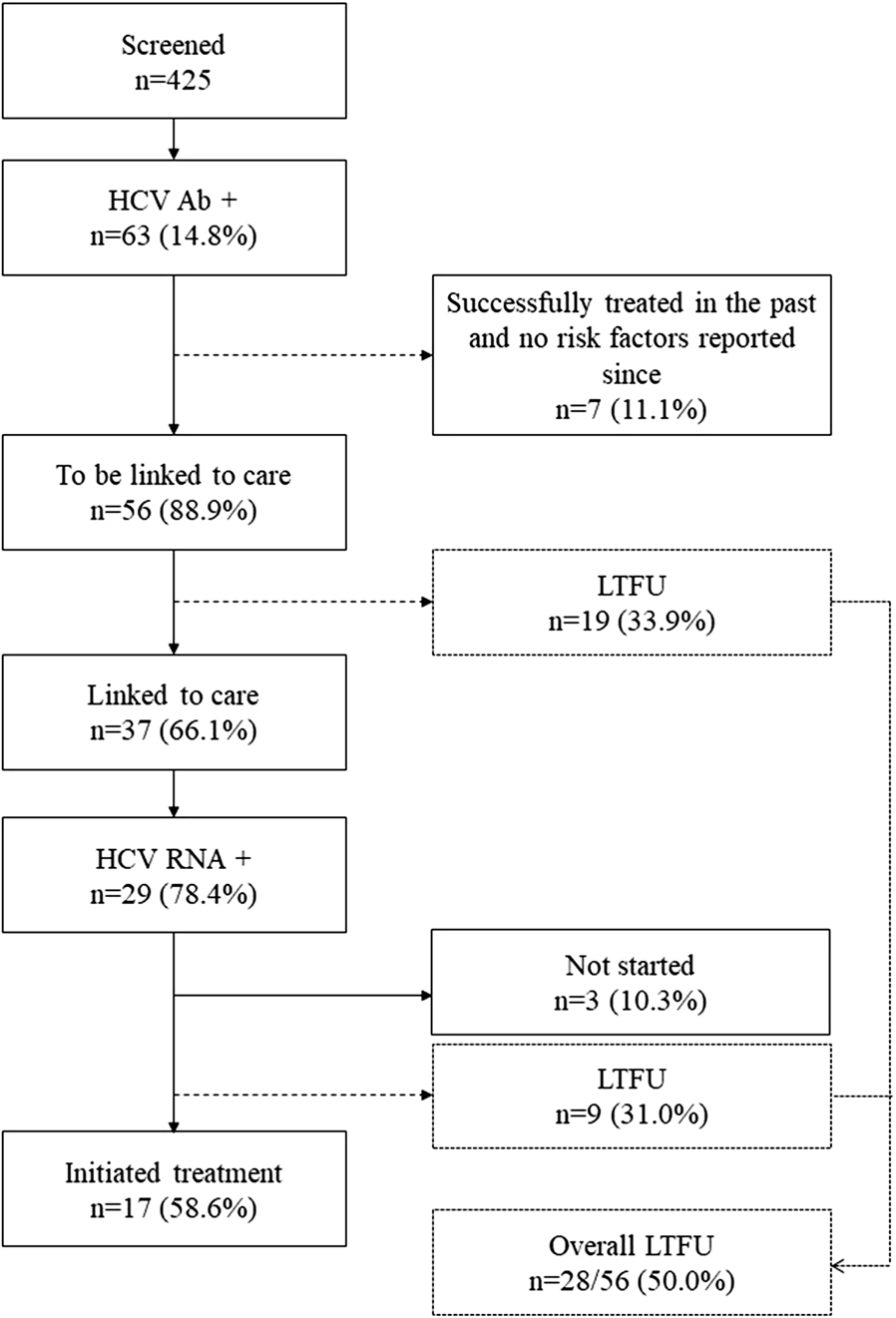
Flowchart for people who use drugs recruited by outreach and linked to care in Belgium

Of the 63 PWUD who tested positive for HCV Ab using a finger prick test, seven (11.1%) had been successfully treated in the past and had not reported any risk factors since. They were therefore not referred to the specialist for further examinations. Of the remaining 56 PWUD, 37 (66.1%) were linked to care and tested by venipuncture for HCV RNA (Fig. 1), of whom 29 (78.4%) were found to have a chronic HCV infection. FibroScan® scores were available for 24 (85.8%) patients. METAVIR scores of *F*0–*F*1, *F*2, *F*3, and *F*4 were found in, respectively, seven (29.2%), seven (29.2%), four (16.7%), and six (20.7%) persons. Genotypes were determined for 28 PWUD. Seven (25.0%) had genotype 1a, three (10.7%) genotype 1b, one (3.6%) genotype 2b, 15 (53.6%) genotype 3, and two (7.1%) genotype 4. Treatment was started in 17 (58.6%) of the 29 HCV RNA positives. Of those who had not yet started, one patient was unwilling, one was too unstable according to the physician, one was taken to prison after screening, and nine were LTFU. Of those who were LTFU, five out of nine had a METAVIR score of F2 or higher, and five stated to have injected drugs in the last 6 months before screening. Overall, we reported a LTFU of 28/56 (50.0%, Fig. 1).

## Discussion

With this large group of PWUD not connected to the OAT program of the center for addiction care in Antwerp or the centralized OAT program of the center in Limburg, this study exceeded the preset sample size. The threshold to be tested was minimal as a non-invasive finger prick was used to test HCV Ab. This is very important as a large part of this population has difficult venous access. An important part of the study group tested positive for HCV Ab (14.8%). Of these HCV Ab positives, 41.2% were unaware that they had ever been in contact with HCV and might have been infected. Our data, therefore, support the elimination of HCV in Europe by substantiating the scientific evidence that not all PWUD are reached through OAT programs, and alternative initiatives need to be implemented to reach these groups [31].

The HCV Ab prevalence in our cohort (14.8%) was many times higher than in the general Belgian population (0.12%) [5]. If we only focus on the PWID in our population, the prevalence is comparable to a Belgian study in PWID and high-risk opiate users (39.2% vs.41.1%) [10]. Looking at the entire screened cohort, 6% were not aware of potential exposure to HCV infection. This is an important finding and contributes to the ‘Belgian Hepatitis C plan.’ One of the action points is to increase the number of HCV-positive patients aware of their diagnosis [32]. Moreover, this finding stresses the importance of screening, not only to identify new cases but also to identify previously known cases and link them back to the cascade of care.

In our study, unstable housing (incarcerated/homeless) last 6 months before inclusion increased HCV risk. Prisoners are more likely to engage in HCV-related risk behavior such as unsterile tattooing, high-risk sexual behavior, and sharing paraphernalia [33, 34]. Worldwide, this has led to an increased prevalence of HCV in prisoners compared to the general population. A review concerning HCV in American homeless people shows an HCV Ab prevalence ranging from 23 to 39% [35]. In a study by Barror et al. [19] in high-risk populations (community addiction, homeless, and prison services) in Ireland, the UK, Romania, and Spain, an HCV Ab prevalence of 37.0% was found. This is slightly higher than our findings and can be explained by the fact that in the study of Barror et al. [19], the proportion that ever injected drugs was higher (44.6%) than in our study population (34.8%).

As expected, HCV odds were increased for those who had ever injected heroin or had injected amphetamines or cocaine in the past 6 months. Moreover, we found a high percentage of people who had injected drugs in the last 6 months. Almost half (49%) of the PWID in our study stated to have injected in the past 6 months before inclusion.

In Belgium, all PWUD can have access to NSP and low threshold OAT even outside an addiction care center (e.g., local pharmacy). Almost 20% of the recent injectors in our study were not connected to NSP, and almost half of the recent opioid users were not connected to an OAT program. These people were informed about NSP and OAT and provided with practical information about the addiction care centers. Studies have shown that OAT is associated with a 50% reduction in the risk of an HCV infection [36]. European studies even show a reduction of 54% in HCV infection risk associated with high NSP coverage [36]. By testing those with ongoing risk factors and informing them, and linking them to harm reduction programs, we can potentially prevent new acute infections. Only one factor seemed to decrease the odds of HCV infection. People who had used cocaine in general in the last 6 months before inclusion showed a significantly lower risk. Although this type of use is not identified as a risk factor in our study, we are aware that sniffing can cause nasal wounds that can lead to HCV transmission when sharing unsterile and contaminated material.

In our study and many others, IV drug use is the most significant risk factor for HCV. Nevertheless, it remains crucial to screen a broader group of PWUD involving not only injecting but also stimulant users. On the one hand, this avoids stigmatizing a subpopulation. On the other hand, if only PWID had been tested in this study, 5/63 (7.9%) PWUD with a potential infection would not have been detected for further follow-up. Moreover, not all PWUD will want or dare to identify themselves as PWID.

To increase the uptake of screening, each participant was provided a ten euro remuneration for participating in the study. International studies show that cash incentives increase the linkage to care and treatment [37, 38]. Despite the effort, linkage to care in this study was lower than expected. A total of 66.1% of PWUD were linked to care. Nevertheless, our findings on linkage to care are in line with Barror et al*.* [19], in which linkage to care ranged from 64 to 89% in those who were referred after a positive HCV RNA test on scene. A study by Poll et al*.* [39] shows that the main reasons for missing an appointment are priority to buy drugs, the cost of travel, or the appointment’s timing. In our study, treatment was initiated in just over half of those linked to care and eligible for treatment. The most common cause for not initiating treatment was the LTFU of the patient. All LTFU patients were active PWID, and 56.0% had stated that they had injected in the 6 months before inclusion. This is a problematic finding because, globally, active PWID form the current heart of the infection. This population should be treated as a priority to contain this epidemic. Multiple methods were used to find and reconnect these PWID. Although all LTFU-PWID in our study were without a trace, we could not confirm the main reason for not initiating treatment. In the past, the strict reimbursement criteria for receiving treatment were probably one of the main causes of LTFU in this population. However, since January 2019, the reimbursement criteria have been adjusted, and every infected person is eligible for treatment despite their degree of fibrosis in Belgium [40, 41]. Besides, five out of nine LTFU had a fibrosis score ≥ F2 and could have been treated in 2018. Additionally, we lack the ability to provide on-site treatment in Belgium. Treatment can only be prescribed and initiated by a hepatologist and is only available in hospital pharmacies, which means that on-site treatment is currently not possible in Belgium. Access to treatment would improve, and patients would receive their medication more easily if treatment could be prescribed by other healthcare professionals and be available in local pharmacies, resulting in less LTFU.

This study has several limitations. During the screening events, participants were only screened for HCV Ab by rapid test. However, an HCV RNA test is necessary to confirm an infection. HCV RNA testing by finger prick using a point of care molecular testing instrument is currently not approved as a diagnostic tool. However, the instrument has recently been validated in a population of Belgian PWUD [42]. Therefore, we were unable to make a diagnosis on scene. Moreover, this may have led to an increased dropout rate because the PWUD had to move to the hospital without the certainty that they were infected. Furthermore, before January 2019, the main reason for not starting treatment was probably Belgium’s strict reimbursement criteria. Since January 2019, the lack of the possibility to offer on-site treatment seems to be the leading cause of not starting treatment. As a result, many PWUD disappear after screening and never reach the hospital for their specialist appointment. In order to proceed to on-site treatment in Belgium, discussions at ministerial level are inevitable. Further, although convenience sampling was used in this study, participants were recruited at different locations to reach a wide range of PWUD and limit selection bias. However, even though we have tried to avoid selection bias as much as possible, it can never be avoided entirely. For example, extremely high-risk PWUD, especially PWID, may not have been included in our study. These results should not be generalized to the entire PWUD population. Finally, the data were derived by means of a face-to-face questionnaire that could have led to a social desirability bias. This could have led to an underreporting of IV drug use. Nevertheless, the PWUD population and more specific the PWIDs are the most important group to find, test, diagnose, and (re-)connect to (low-threshold) health care as they are the heart of the HCV epidemic.

## Conclusions

We were able to screen a group of hard-to-reach PWUD, and although Belgium is a country with low-threshold access to addiction care (e.g., local pharmacy), not everyone was linked to addiction care. An important part of the study group tested positive for HCV Ab. In more than half of the chronically infected, treatment could be started. This study provides critical local data on the current epidemic in a high-income country and could be valuable for other regions with similar epidemiological and healthcare systems. Micro-elimination is necessary to achieve the WHO goals, but it remains critical to screen and inform a broader group of PWUD involving not only injecting but also stimulant users. Also, it is essential to actively seek out high-risk groups that remain under the radar of primary care, as demonstrated in this study. If we want to achieve the WHO goals by 2030 in Belgium, we urgently need a nationally implemented screening and treatment strategy that preferably allows for testing with point of care HCV RNA testing, fibrosis staging (mobile FibroScan®), and on-site treatment.

## Supplementary Information

**Additional file 1.** **A1.** Formula for sample size calculation.

**Additional file 2. **. **A2.** Univariate generalized linear mixed models to investigate the association between different risk factors and hepatitis C antibodies.

## Data Availability

The data supporting the conclusions of this article are included within the article and its additional files.

## Abbreviations

HCV: Hepatitis C virus
WHO: World Health Organization
PWUD: People who use drugs
Ab: Antibody
PWID: People who inject drugs
OAT: Opioid agonist treatment
NSP: Needle syringe program
LTFU: Lost to follow-up
IV: Intravenous
GLMM: Generalized linear mixed model
CI: Confidence interval
OR: Odds ratio
AOR: Adjusted odds ratio
