# Supplementary figures and images for: Identification and treatment of viral hepatitis C in persons who use drugs: a prospective, multicenter outreach study in Flanders, Belgium
Source: Harm Reduct J. 2021 May 17;18:54. doi: 10.1186/s12954-021-00502-7 (PMC8130277; doi:10.1186/s12954-021-00502-7)

**Additional file 1**


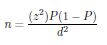
**A1. Formula for sample size calculation**

Supplement: Supplementary file 1 — Additional file 1. A1. Formula for sample size calculation. [file 12954_2021_502_MOESM1_ESM.docx]
