# Supplementary material for: Identification and treatment of viral hepatitis C in persons who use drugs: a prospective, multicenter outreach study in Flanders, Belgium
Source: Harm Reduct J. 2021 May 17;18:54. doi: 10.1186/s12954-021-00502-7 (PMC8130277; doi:10.1186/s12954-021-00502-7)
Supplement: Supplementary file 2 — Additional file 2. . A2. Univariate generalized linear mixed models to investigate the association between different risk factors and hepatitis C antibodies. [file 12954_2021_502_MOESM2_ESM.docx]

**Additional file 2**

**A2. Univariate generalized linear mixed models to investigate the association between different risk factors and hepatitis C antibodies**

| **Risk factor** |  | **n#** | **N** | **% (n#/N)** | ***p*-value** | **OR (95% CI)** |
| --- | --- | --- | --- | --- | --- | --- |
| ***Demographics*** | | | | | | |
| Year of birth | < 1955  1955 – 1974  >1974 | 1  36  26 | 10  153  259 | 10.0  23.5  10.0 | ref  .369  .976 | 2.637 (0.460 – 49.850)  0.968 (0.169 – 18.298) |
| Gender | Male  Female | 51  12 | 335  90 | 15.2  13.3 | .692  ref | 1.147 (0.598 – 2.355) |
| Birth country | Belgium  Other | 53  10 | 333  91 | 15.9  11.0 | ref  .161 | 0.590 (0.268 – 1.191) |
| Birth country mother | Belgium  Other | 45  18 | 283  142 | 15.9  12.7 | ref  .291 | 0.724 (0.389 – 1.300) |
| Birth country father | Belgium  Other | 43  20 | 283  142 | 15.2  14.1 | ref  .646 | 0.872 (0.478 – 1.547) |
| Main income source | Employment  Unregistered  Allowance  Pension  No income | 4  2  46  2  9 | 87  21  254  13  47 | 4.6  9.5  18.1  15.4  19.1 | ref  .432  **.006**  .153  **.014** | 2.053 (0.265 – 11.682)  4.477 (1.733 – 15.277)  3.745 (0.479 – 21.730)  4.764 (1.434 – 18.648) |
| Level of education | Primary school  High school  College / University | 24  29  10 | 152  206  66 | 15.8  14.1  15.1 | ref  .651  .886 | 0.873 (0.485 – 1.582)  0.943 (0.406 – 2.058) |
| Housing situation | Owned / rented  Residential / family / friends  Prison / homeless / other | 26  20  17 | 225  122  76 | 11.6  16.4  22.4 | ref  .207  **.022** | 1.501 (0.779 – 2.810)  2.205 (1.054 – 4.315) |
| Sexual preference | Hetero  Homosexual  Bisexual | 54  2  5 | 382  9  27 | 14.1  22.2  18.5 | ref  .603  .535 | 1.538 (0.222 – 6.748)  1.379 (0.446 – 3.547) |
| ***Risk behavior*** | | | | | | |
| Number of unsafe partners | Never  1 – 10  >10 | 13  19  30 | 112  154  156 | 11.6  12.3  19.2 | ref  .881  .145 | 1.059 (0.501 – 2.300)  1.740 (0.843 – 3.730) |
| Ever incarcerated | Yes  No | 53  10 | 227  194 | 23.4  5.2 | **<.001**  ref | 5.557 (2.847 – 11.933) |
| Tattoo | None  Safe  Potentially unsafe | 14  25  24 | 146  174  102 | 9.6  14.4  23.5 | ref  .197  **.002** | 1.582 (0.798 – 3.252)  3.161 (1.542 – 6.715) |
| Alcohol abuse | Never  Past  Present | 19  17  27 | 175  126  122 | 10.9  13.5  22.1 | ref  .481  **.013** | 1.287 (0.633 – 2.598)  2.302 (1.201 – 4.467) |
| ***Drug use*** | | | | | | |
| Age first drug use |  |  |  |  | .872 | 0.997 (0.960 – 1.029) |
| Drugs used last 6 months | No  Yes | 7  56 | 102  323 | 6.8  17.3 | ref  **.014** | 2.800 (1.308 – 6.946) |
| Manner: oral | Yes  No | 51  12 | 268  157 | 19.0  7.6 | **.001**  ref | 3.052 (1.606 – 6.240) |
| Manner: sniffing | Yes  No | 52  11 | 310  115 | 16.8  9.6 | .060  ref | 1.940 (1.006 – 4.062) |
| Manner: smoking | Yes  No | 61  2 | 406  19 | 15.0  10.5 | .650  ref | 1.416 (0.386 – 9.128) |
| ***Drugs used ever*** | | | | | | |
| Amphetamines | No  Yes | 10  53 | 144  281 | 6.9  18.9 | ref  **.001** | 3.242 (1.653 – 6.990) |
| Cocaine | No  Yes | 4  59 | 111  314 | 3.6  18.8 | ref  **.001** | 6.254 (2.490 – 21.007) |
| Heroin | No  Yes | 9  54 | 240  185 | 3.8  29.2 | ref  **<.001** | 10.640 (5.326 – 23.735) |
| ***Drugs used in the last 6 months*** | | | | | | |
| Amphetamines | No  Yes | 32  31 | 282  143 | 11.3  21.7 | ref  **.006** | 2.138 (1.238 – 3.690) |
| Cocaine | No  Yes | 36  27 | 281  144 | 12.8  18.8 | ref  .128 | 1.534 (0.877 – 2.658) |
| Heroin | No  Yes | 31  32 | 328  97 | 9.5  33.0 | ref  **<.001** | 4.682 (2.663 – 8.266) |
| ***IV drug use*** | | | | | | |
| IV drug use | Never  Ever  Last 6 months | 8  20  35 | 282  70  73 | 2.8  27.8  47.9 | ref  **<.001**  **<.001** | 15.324 (6.433 – 39.908)  31.278 (14.089 – 77.480) |
| Frequency IV drug use | Never  < once a week  >once a week  Daily | 28  11  14  10 | 350  30  24  21 | 8.0  36.7  58.3  47.6 | ref  **<.001**  **<.001**  **<.001** | 6.658 (2.819 – 15.249)  16.100 (6.623 – 40.648)  10.455 (4.038 – 26.982) |
| Shared paraphernalia | Never  Ever  Last 6 months | 22  34  7 | 335  69  21 | 6.6  49.3  33.3 | ref  **<.001**  **<.001** | 13.801 (7.341 – 26.579)  7.049 (2.453 – 18.927) |
| OAT | Not applicable  Yes  No | 9  23  31 | 227  65  133 | 4.0  35.4  23.3 | ref  **<.001**  **<.001** | 13.095 (5.819 – 31.847)  7.400 (3.526 – 17.059) |
| NSP | Not applicable  Yes  No | 43  15  5 | 347  65  13 | 12.4  23.1  38.5 | ref  **.008**  **.008** | 2.629 (1.268 – 5.315)  5.007 (1.428 – 16.214) |
| ***IV drug use ever*** | | | | | | |
| Amphetamines | No  Yes | 23  40 | 338  87 | 6.8  46.0 | ref  **<.001** | 11.627 (6.453 – 21.447) |
| Cocaine | No  Yes | 19  44 | 329  96 | 5.8  45.8 | ref  **<.001** | 13.940 (7.640 – 26.355) |
| Heroin | No  Yes | 15  48 | 322  103 | 4.7  46.6 | ref  **<.001** | 18.154 (9.678 – 35.891) |
| ***IV drug use in the last 6 months*** | | | | | | |
| Amphetamines | No  Yes | 37  26 | 375  50 | 9.9  52.0 | ref  **<.001** | 9.843 (5.141 – 19.049) |
| Cocaine | No  Yes | 41  22 | 386  39 | 10.6  56.4 | ref  **<.001** | 10.839 (5.343 – 22.412) |
| Heroin | No  Yes | 41  22 | 388  37 | 10.6  59.5 | ref  **<.001** | 12.413 (6.027 – 26.275) |

Abbreviations: OR: odds ratio; IV: intravenous; OAT: opiate agonist therapy; NSP: needle syringe program; ref: reference
